# Effects of Casein Kinase 2 Alpha 1 Gene Expression on Mice Liver Susceptible to Type 2 Diabetes Mellitus and Obesity

**DOI:** 10.7150/ijms.37110

**Published:** 2020-01-01

**Authors:** Yu-Ching Lan, Yeh-Han Wang, Hsin-Han Chen, Sui-Foon Lo, Shih-Yin Chen, Fuu-Jen Tsai

**Affiliations:** 1 Department of Health Risk Management, China Medical University, 40402 Taichung, Taiwan;; 2 Department of Anatomical Pathology, Taipei Institute of Pathology, School of Medicine, National Yang-Ming University , 11221 Taipei, Taiwan;; 3 Division of Plastic and Reconstructive Surgery, China Medical University Hospital, 40402 Taichung, Taiwan;; 4 School of Chinese Medicine, China Medical University, 40402 Taichung, Taiwan;; 5 Genetics Center, Medical Research, China Medical University Hospital, 40447 Taichung, Taiwan;; 6 Department of Medical Genetics, China Medical University Hospital, 40447 Taichung, Taiwan, R.O.C.

**Keywords:** +*Lepr*^db^ / +*Lepr*^db^ mice, T2DM, Casein Kinase 2 Alpha 1 (CSNK2A1).

## Abstract

Diabetes mellitus (DM) is a chronic disease found worldwide. Notably, BKS.Cg- Dock7^m^* +/+* Lepr^db^/JNarl mice are useful animal models for studying type 2 diabetes mellitus (T2DM). In this study, we investigated casein kinase 2 alpha 1 (CSNK2A1) gene and protein expression in the liver tissues of mice at different ages (4, 16, and 32 weeks) using real-time quantitative polymerase chain reactions, western blotting, immunohistochemistry, and enzyme-linked immunosorbent assay. Our data paved the way for exploring BKS.Cg- Dock7^m^ +/+ Lepr^db^/JNarl in the mouse model by demonstrating a significant increase in gene and protein expression in T2DM (+Lepr^db^/+Lepr^db^) mouse liver when compared to control (+Dock7^m^/+Dock7^m^) mouse liver. We also observed that CSNK2A1 protein level in the serum of T2DM patient group was higher than that of the control group, although the data was not statistically significant. Based on our findings, we can now understand the role of *CSNK2A1* gene upregulation when encountering T2DM pathologies.

## Introduction

The protein kinase casein kinase 2 (CSNK2 or CK2) is a serine/threonine protein kinase that is expressed in most cell types 1. CSNK2 phosphorylates various proteins that deal with cell cycle regulation, cell survival, cell morphology, cell metabolism, tumorigenesis, and cancer cell invasiveness 2. It is composed of 2 large catalytic subunits, CK2α (44 kDa) and CK2α' (36 kDa), and 2 small non-catalytic CK2β subunits (25 kDa). CK2α is a human enzyme encoded by the CSNK2A1 gene 3. Notably, CSNK2A1 has been found to be highly expressed in a wide variety of cancers. At the transcriptional and/or protein level, CK2 overexpression, particularly in the α catalytic subunit (CK2α or CSNK2A1), has been observed in many cancers 4. A growing body of evidence shows that the insulin signaling system plays a key role in cancer development and progression. CK2 also plays an important role in the regulation of carbohydrate metabolism 5. Hence, CK2 has recently been recognized as a “master kinase” that is involved in many important cellular processes by controlling the activity of several other kinases 1.

G protein-coupled receptors (GPCRs) regulate the enzymatic activity of virtually all cell types, including pancreatic β-cells. Furthermore, β-cell M3 muscarinic receptors (M3Rs) play an important role in maintaining proper whole-body glucose homeostasis 6. Similar to other GPCRs, various kinases modulate M3R activity via phosphorylation. When insulin is released, CK2-dependent phosphorylation of β-cell M3Rs significantly damages M3R-mediated increases in protein expression. The physiological relation between CK2 phosphorylation and GPCRs suggests that the kinases acting on β-cell GPCRs may be considered as targets for therapy. CK2 inhibition has also been shown to strongly enhance M3R-stimulated insulin secretion in isolated pancreatic islets or cultured β-cells 7. However, studies about CSNK2A1 expression in liver cells are severely lacking.

Diabetes mellitus (DM) is a complex disease that occurs due to absolute or relative insulin deficiency. Lifestyle modifications and nutritional adjustments are some of the best ways to prevent and treat type 2 diabetes mellitus (T2DM) 8. Notably, genetic factors have also been shown to play an important role in DM and have been suggested as new ways of combating T2DM 8,9. In this study, we show that CSNK2A1 is involved in the regulation of glucose stimulated insulin secretion (GSIS). A previous study demonstrated that increased CSNK2A1 activity is correlated with enhanced insulin secretion and consistent with the MS-based proteomic profile, a significant reduction of CK2A levels was observed in NOD (non-obese diabetic mice) diabetic islets 10. In previous cancer studies, CSNK2A1 overexpression at the transcriptional and/or protein level was observed in breast and liver cancer samples 4,11,12. Due to a lack of studies regarding CSNK2A1 expression at the genomic and protein levels in T2DM and obesity, we used the T2DM and obesity mouse model in this study to investigate the impact of CSNK2A1 on T2DM and obesity. Here, we showed strong evidence of the role of CSNK2A1 gene and protein expression in obesity and T2DM. By using an animal model, we demonstrated CSNK2A1 gene and protein expression in the liver tissue of different mouse groups to better understand the role in the pathological features of T2DM.

## Materials and methods

### Animal model

Twenty-four 4-week-old male BKS.Cg- Dock7^m^ +/+ Lepr^db^/JNarl mice along with control (+Dock7^m^/+Dock7^m^; n = 12) and T2DM (+Lepr^db^/+Lepr^db^; n = 12) mice were obtained from the National Laboratory Animal Center (NLAC) in Taiwan. All animals were raised in individual cages and placed in rooms that had a relative humidity of 50-70%, constant temperature of 22-25 °C, and 12 h light/dark cycles. There were 6 groups in the study: 3 control groups at 4, 16, and 32 weeks; and 3 T2DM groups at 4, 16, and 32 weeks. Our study was reviewed and approved by the Institutional Animal Care and Use Committee (IACUC) of China Medical University (IACUC: 2016-221).

### Real-time quantitative polymerase chain reaction

The RNeasy Mini Kit (Qiagen, Germantown, MD, USA) was used to isolate total RNA from ground liver tissue of control and T2DM mice. The SuperScript First-Strand Synthesis Kit (Invitrogen) was used for cDNA transcription. To study gene expression, real-time quantitative polymerase chain reactions (RT-qPCRs) were performed using TaqMan assays (Applied Biosystems, CA, USA) for murine CSNK2A1 (NM_007788.3) on a Prism 7900HT Sequence Detection System (Applied Biosystems). The target gene expression levels were normalized to mice glyceraldehyde-3-phosphate dehydrogenase (GAPDH; M32599).

### Western blot analysis

In this study, murine anti-CSNK2A1 (GTX107897) monoclonal antibodies were used to detect CSNK2A1 via western blotting procedures described previously 13. Briefly, frozen liver tissue samples were homogenized with 3 volumes of 10 mM ice-cold phosphate buffer (pH 7.0) containing 1 mM EDTA, 0.25 M sucrose, 1 mM sodium azide, and 0.1 mM phenylmethylsulfonyl fluoride. Samples were then centrifugated at 20,000 ×*g* for 30 min at 4 ℃. Protein concentrations were measured using BCA assay (Pierce Biotechnology, Rockford, IL, USA) with albumin as the standard. The tissue lysates were subjected to denaturing electrophoresis via 10% SDS-polyacrylamide gel, electro-transferred to PVDF membranes, and immune-stained with CSNK2A1 and β-actin antibodies. The bands were analyzed using the Enhanced Chemiluminescence Kit (Amersham, Buckinghamshire, UK).

### Immunohistochemistry analysis

CSNK2A1 protein expression was determined via immunohistochemistry (IHC) analysis using paraffin-embedded liver sections. Anti-CSNK2A1 IHC staining was carried out using the LsAB Kit (DAKO, Glostrup, Denmark). All tissue sections were de-waxed, treated with Proteinase K enzyme, and the endogenous peroxidase activity was blocked by incubating with 3% hydrogen peroxide for 10 min. After washing with phosphate-buffered saline (PBS; pH 7.6) for 5 min, the slides were incubated with anti-CSNK2A1 antibodies (GTX84369; GeneTex, Hsinchu, Taiwan) for 30 min at 37 ℃, followed by rabbit anti-rat antibodies and a goat anti-rabbit HRP polymer for 15 min. The immunocomplexes were visualized using DAB solution (DAKO) for 5 min. Samples were washed with PBS (pH 7.6) in order to perform all the necessary steps 14,15,16.

### Enzyme linked immunosorbent assay (ELISA) of CSNK2A1 protein levels in mouse liver and human serum

Mouse liver tissue were minced after weighing and homogenized in PBS with a glass homogenizer on ice. The homogenates were then centrifuged at 5,000 ×*g* for 5 min and supernatants were collected to measure protein concentrations at room temperature. CSNK2A1 protein levels in mouse liver and human serum were determined using a suite of commercial kits (Catalogue No: EM7856 for mouse and EH1138 for human; Wuhan Fine Biological Technology Co., Hubei, China) according to the manufacturer's instructions.

### Biochemical assessment

Glutamic-pyruvic transaminase (GPT) levels were determined using a Spotchem EZ analyzer that uses carrier strips (Menarini Diagnostics, Wokingham, UK). Samples were run on the instrument using previously described standard methodologies 17. Analyses and operations were based on the manufacturer's standard processes and all samples were run by the same technician.

### Patients and sample collection

In this study, 2 male and 6 female patients (ranging from 45-65 years old) that fulfilled the diagnostic criteria of obesity (BMI > 27) and T2DM were enrolled at the China Medical University Hospital in Taiwan between Aug 2014 and July 2015. Age- and gender-matched unrelated healthy controls were also obtained from the general population at the same hospital during the same time period. Serum samples were collected for ELISA. The protocols were approved by the ethical committee of China Medical University Hospital (No. CMUH103-REC2-071). Informed consent was obtained from all individuals enrolled in the study.

### Statistical analysis

Data is expressed as mean ± standard error of 3 independent experiments. Statistical comparison between the test and control groups was performed using the Student's *t*-test. P < 0.05 was considered to be statistically significant.

## Results

**Figure 1:**
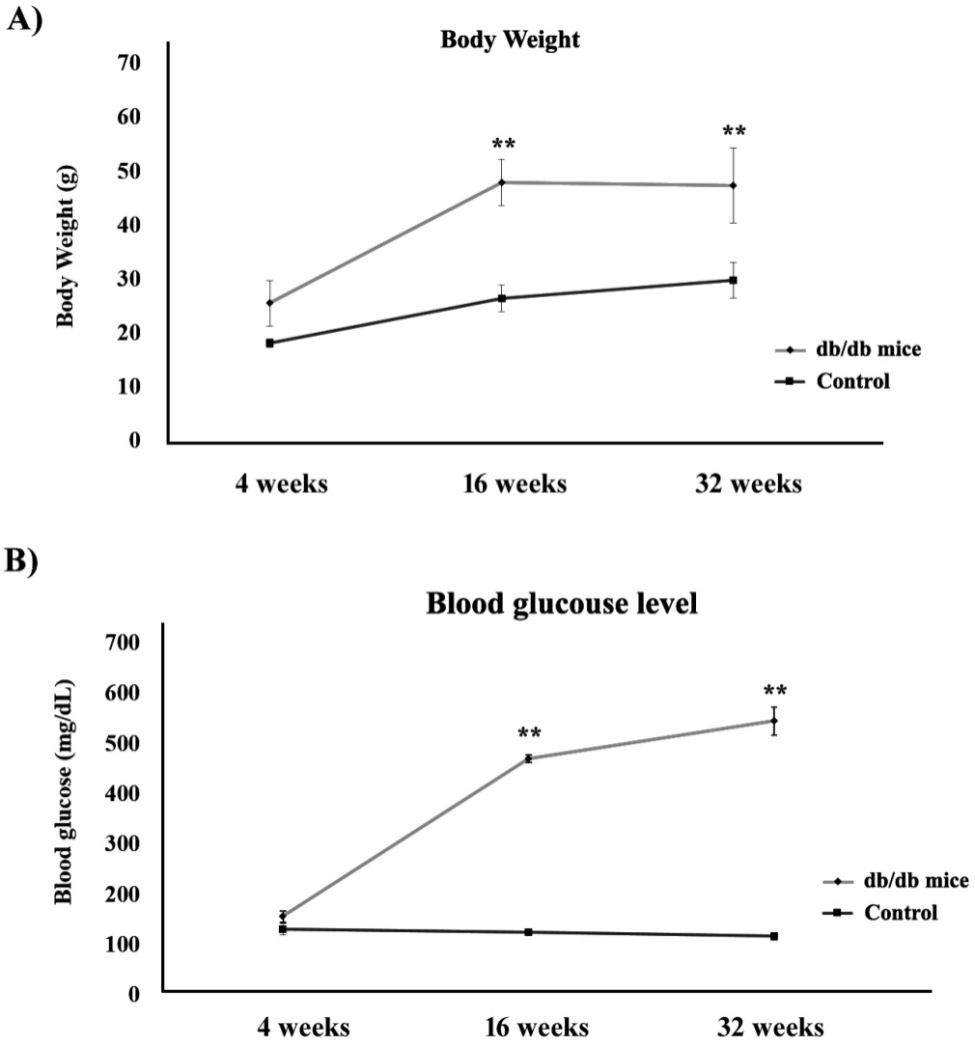
Estimation of (A) body weight and (B) blood glucose levels in control (■) and T2DM (●) mouse models (**, P < 0.05).

Figure 1A shows the body weights of the control (+Dock7^m^/+Dock7^m^) and T2DM (+Lepr^db^/+Lepr^db^) mouse models over 4, 16, and 32 weeks. Our data indicated that the body weights in the T2DM mouse groups increased significantly when compared to the control groups, thus doubling up as an obesity animal model (P < 0.05). We also observed that blood glucose levels in the T2DM mouse groups increased significantly from 4 to 32 weeks (P < 0.05) i.e., more than 500 mg/dL of blood glucose was detected in the T2DM mouse groups at 32 weeks (Figure 1B).

**Figure 2:**
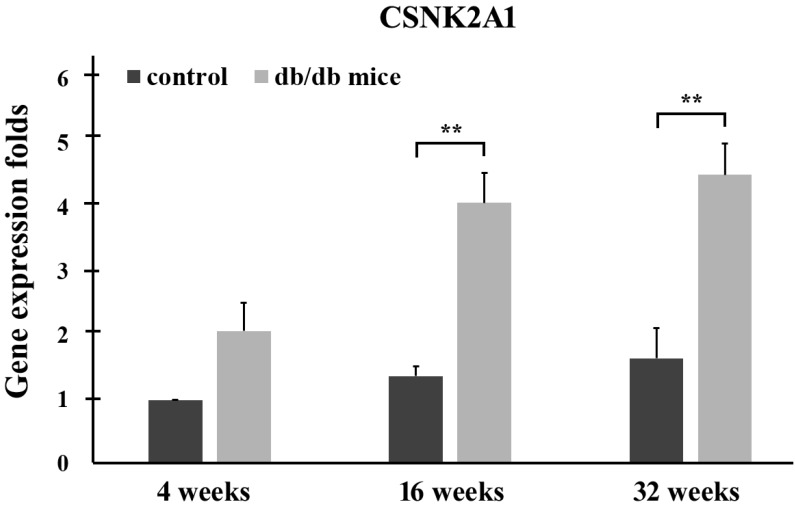
Quantitative real-time reverse transcription polymerase chain reaction analysis of the CSNK2A1 gene in the liver tissues of control (+Dock7^m^/+Dock7^m^) and T2DM (+Lepr^db^/+Lepr^db^) mice at 4, 16, and 32 weeks. Gene expression data of CSNK2A1 was calculated after normalizing against GADPH (**, P < 0.05).

Control (+Dock7^m^/+Dock7^m^) and T2DM (+Lepr^db^/+Lepr^db^) mice were individually sacrificed at 4, 16, and 32 weeks. RNA from the liver tissues was then extracted for quantitative real-time reverse transcription polymerase chain reaction analysis. The qPCR data in Figure 2 shows CSNK2A1 gene expression in the liver tissue of mice aged 4, 16, and 32 weeks. The results showed that CSNK2A1 gene expression in the liver tissue of +Lepr^db^/+Lepr^db^ mice (T2DM) was significantly higher than that in the liver tissue of +Dock7^m^/+Dock7^m^ mice (control) (P < 0.05; Figure 2). Therefore, the results suggest upregulated CSNK2A1 gene expression in T2DM mice.

**Figure 3:**
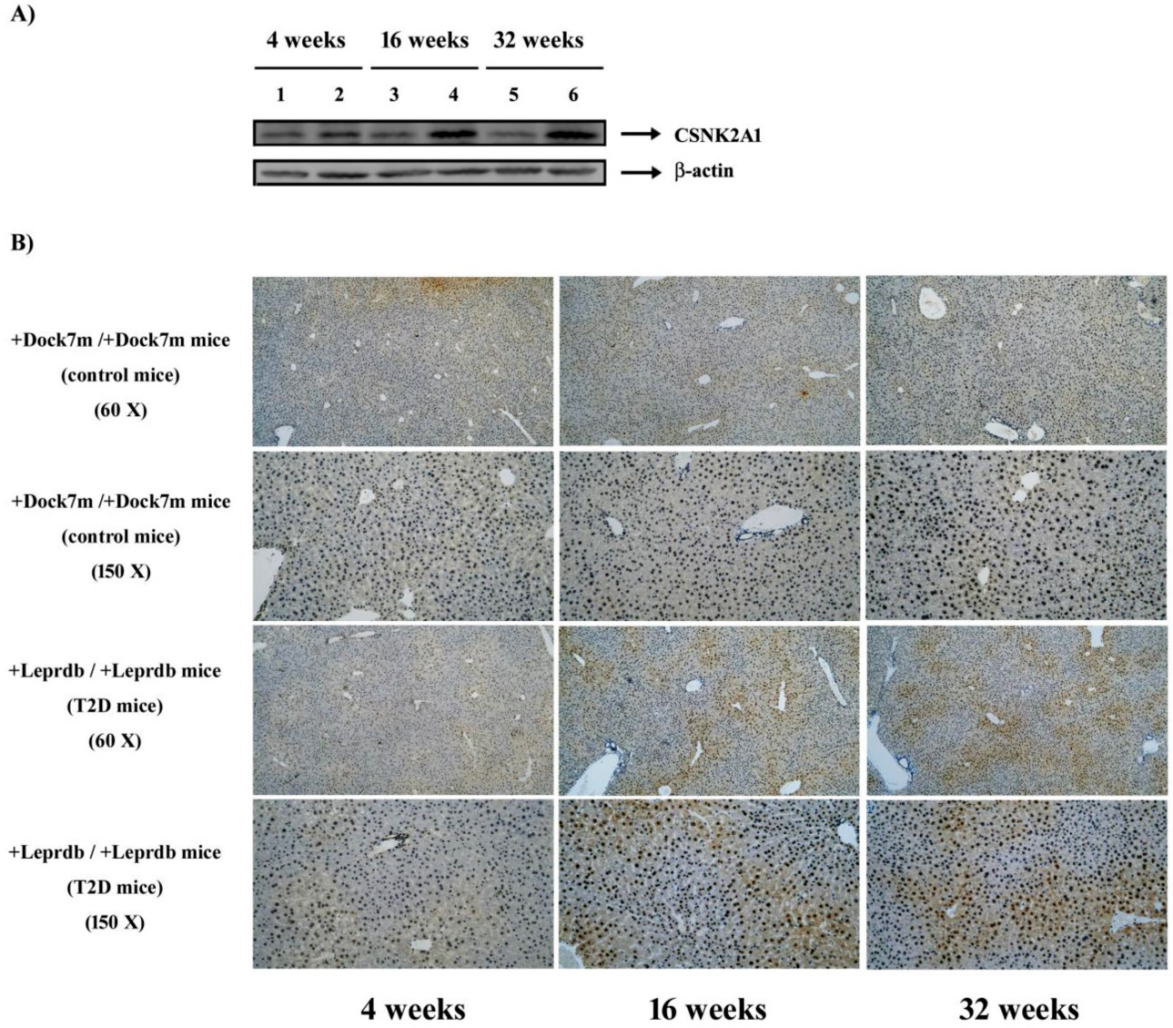
** (A)** Western blot analyses of CSNK2A1 protein expression in the liver tissues of control (+Dock7^m^/+Dock7^m^; lanes 1, 3, and 5) and T2DM (+Lepr^db^/+Lepr^db^; lanes 2, 4, and 6) mice at 4, 16, and 32 weeks, respectively. **(B)** Representative IHC-processed CSNK2A1 expression in T2DM mice (+Lepr^db^/+Lepr^db^) is shown. Progression of the mouse models for T2DM is also shown. Liver tissues were excised, fixed, embedded, and sectioned for IHC staining as described in the Materials and Methods.

Control and T2DM mice were individually sacrificed at 4, 16, and 32 weeks. Mouse liver tissues were homogenized and 20 μg of protein was analyzed via western blotting using anti-CSNK2A1 and β-actin antibodies. Figure 3A shows representative blots for control (lanes 1, 3, and 5) and T2DM (lanes 2, 4, and 6) mice at 4, 16, and 32 weeks, respectively. Liver tissues were also excised, fixed, embedded, and sectioned for IHC staining. Figure 3B shows IHC-processed CSNK2A1 protein expression in the liver tissues of mice aged 4, 16, and 32 weeks. The results showed that CSNK2A1 protein expression in the liver tissue of +Lepr^db^/+Lepr^db^ mice (T2DM) was significantly higher than that in the liver tissue of +Dock7^m^/+Dock7^m^ mice (control) (Figure 3). Therefore, the results suggest upregulated CSNK2A1 protein expression in T2DM mice.

**Figure 4:**
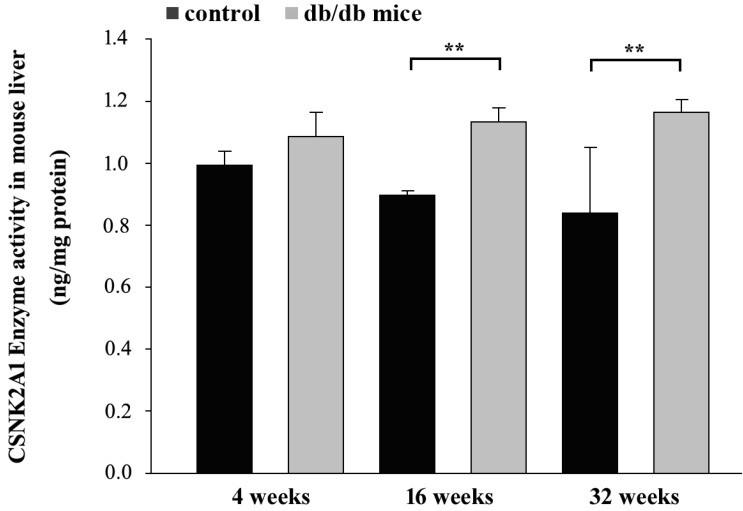
ELISA-mediated measurement of CSNK2A1 protein levels in the liver tissues of control (+Dock7^m^/+Dock7^m^) and T2DM (+Lepr^db^/+Lepr^db^) mice at 4, 16, and 32 weeks. **, P < 0.05 for the indicated comparisons.

Control and T2DM mice were individually sacrificed at 4, 16, and 32 weeks. Proteins from the liver tissues were then extracted for ELISA. The ELISA data in Figure 4 shows CSNK2A1 protein levels in the liver tissue of mice aged 4, 16, and 32 weeks. The results showed that CSNK2A1 protein levels in the liver tissue of +Lepr^db^/+Lepr^db^ mice (T2DM) were significantly higher than that in the liver tissue of +Dock7^m^/+Dock7^m^ mice (control) at 16 and 32 weeks (P < 0.05; Figure 4). Therefore, the results suggest upregulated CSNK2A1 protein levels in T2DM mice.

**Figure 5:**
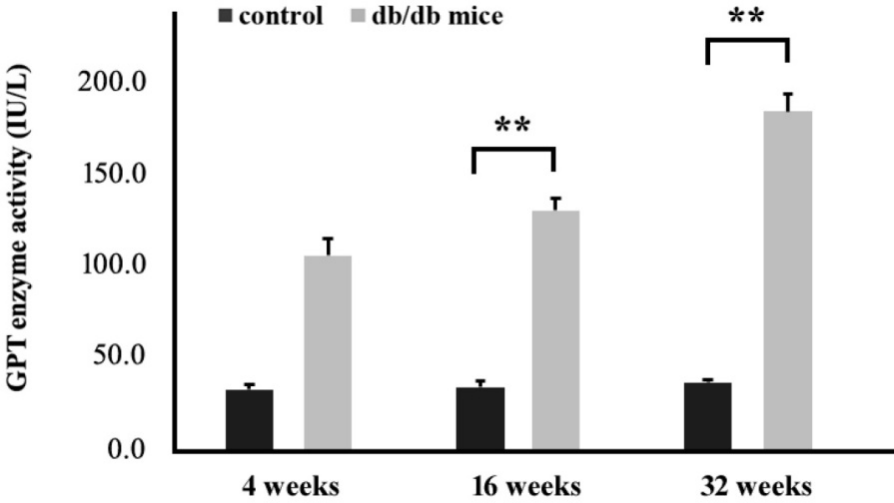
Measurement of GPT activity in the blood of control (+Dock7^m^/+Dock7^m^) and T2DM (+Lepr^db^/+Lepr^db^) mice at 4, 16, and 32 weeks. **, P < 0.05 for the indicated comparisons.

Next, mice were sacrificed and blood samples were collected from each mouse in order to measure serum glutamic pyruvic acid transaminase (GPT) activity using a Spotchem EZ analyzer that uses carrier strips. Our data indicated that high blood glucose levels in the T2DM group (+Lepr^db^/+Lepr^db^) significantly increased serum GPT activity when compared to the control group (+Dock7^m^/+Dock7^m^) (P < 0.05; Figure 5).

**Figure 6:**
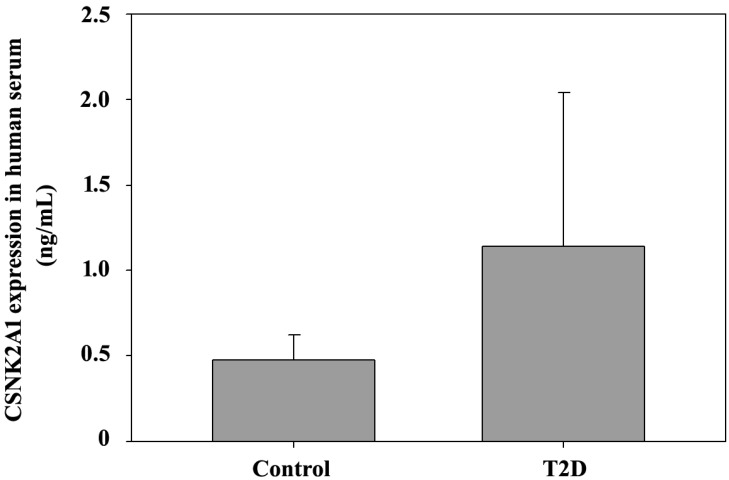
ELISA-mediated measurement of CSNK2A1 protein levels in the human serum of T2DM patients and controls.

Finally, ELISA was used to detect CSNK2A1 expression in T2DM patients with obesity. Serum samples were collected from 8 T2DM patients and 3 non-T2DM patients (BMI > 27). The ELISA data in Figure 6 shows CSNK2A1 protein levels in human serum. The results showed that CSNK2A1 protein levels in the serum of the T2DM patient group was higher than that in the serum of the control group, although the data was not statistically significant (Figure 6). Therefore, the results suggest upregulated CSNK2A1 protein levels in T2DM patients.

## Discussion

To our knowledge, this is the first systematic study that used the time serial animal model spanning the early to the late DM state to investigate the association between CSNK2A1, obesity, and T2DM from the genomic to the phenotypic level in the liver. Our animal model results generally demonstrated diabetes and obesity in animals by showing a significant increase in body weight and blood glucose levels in the T2DM mouse group 18,19. Furthermore, biochemical assessment of GPT in T2DM mice also showed a significant increase over time even though its level was significantly high in the control group, which showed low liver functionality in the T2DM mouse group 20,21.

In this study, we investigated CSNK2A1 gene and protein expression in the liver tissues of mice at different ages (4, 16, and 32 weeks) via a systematic method using real-time qPCRs, western blot assays, IHC, and ELISA. Our data demonstrated a significant increase in gene and protein expression in the liver tissues of T2DM (+Lepr^db^/+Lepr^db^) and control (+Dock7^m^/+Dock7^m^) mice. We also observed similar results in the serum samples of T2DM patients with obesity; however, the interpretation was limited by the small sample size used in this study. Hence, further studies are required to explore CSNK2A1 protein levels in T2DM patients with obesity using a larger sample size. However, our data paved the way to exploring the BKS.Cg- Dock7^m^ +/+ Lepr^db^/JNarl mouse model. Using our results, we can now better understand the role of CSNK2A1 gene expression in T2DM pathological features.

Notably, CSNK2A1 transcripts and proteins are reported to be upregulated in many kinds of cancers 4,22, such as multiple myeloma 23, breast cancer 11, and liver cancer 12. Moreover, poor patient survival rates have been correlated with CSNK2A1 overexpression 24. In addition, CSNK2A1 gene overexpression was observed in large HCC patient cohorts 25. A study by Zhang et al. found that CSNK2A1 was significantly overexpressed (greater than a 2-fold increase) at the mRNA level in HCC tissues, the expression of which was also reflected at the CSNK2A1 protein level 12. In this study, the strong expression of the CSNK2A1 gene in the liver tissues of T2DM mice not only showed the same results as the previously mentioned study, but also showed a high expression level in β-cells 7. The CSNK2A1 gene was further observed to be expressed at a 1.2-fold higher value in human β-cells isolated from T2DM patients when compared to non-diabetic individuals 26. Thus, these results provide sufficient evidence to associate CSNK2A1 gene expression with T2DM. Furthermore, CSNK2A1 protein expression in the diabetic group was also significantly higher than the control group. CSNK2A1 inhibition in pancreatic β-cells, knockdown of CSNK2A1 expression, and/or genetic deletion of CSNK2A1 β-cells in mutant mice were previously shown to negatively regulate insulin secretion *in vivo* and *in vitro* 7. An association between CSNK2A1 and the atypical NF-κB pathway was also confirmed previously 27. Notably, insulin/IGF-1 acts via 2 mechanisms (AKT and mTOR signaling) to activate NF-κB 28. Combining these findings with our results, it is possible that CSNK2A1 plays an important role in T2DM development. Therefore, the mechanisms of insulin sensitivity and glucose homeostasis warrant further investigation.

Notably, compared to our study that showed upregulated CSNK2A1 gene and protein expression, the previous study used a non-obese diabetic (NOD) mouse model that resulted in the downregulation of protein levels in the islet cells of the diabetic group when compared to the islet cells of the non-diabetic group 10. The difference between our animal model (Obese Diabetes Mouse Model) and the Sacco animal model (Non-Obese Diabetes Model) resided in the fact that our model showed a possible correlation between CSNK2A1 and the obesity mechanism and regulation. Furthermore, we also presented time serial changes for obesity and diabetic parameters such as BMI, blood glucose, serum GPT enzyme activity, and CSNK2A1 gene and protein expression in this study. Previous studies showed that a significant increase in the level of serum GPT was noted in STZ-diabetic mice 29. In addition, a high level of serum GPT was observed in diabetic mice with fatty liver as compared to the control 30. Serum GPT is commonly used to detect non-alcoholic fatty liver disease and has been associated with increased risk of T2DM 31. Therefore, all these evidences showed that the CSNK2A1 protein possibly played an important role in the obesity mechanism.

In conclusion, our study showed the upregulation of CSNK2A1 gene expression and protein expression in T2DM mice, which confirmed the relationship between CSNK2A1 and T2DM. Altogether, our results indicate that CSNK2A1 plays an important role in T2DM and obesity regulation. To our knowledge, this is the first study that used the time serial animal model to investigate the association between CSNK2A1 and obesity and T2DM disease progression from the gene expression level to the phenotypic level. However, further studies are required to understand the mechanisms of CSNK2A1 at the genomic and protein levels and to understand its association with pancreatic β-cell-mediated T2DM and obesity.

## Abbreviations

T2DM: type 2 diabetes mellitus
CSNK2A1: casein kinase 2 alpha 1
qPCRs: quantitative polymerase chain reactions
IHC: immunohistochemistry
ELISA: enzyme-linked immunosorbent assay
GPT: glutamic-pyruvic transaminase
M3Rs: β-cell M3 muscarinic receptors
GAPDH: glyceraldehyde-3-phosphate dehydrogenase
PBS: phosphate buffered saline
